# Olive Components (Biophenols or Polyphenols) in Neurodegenerative Disease Models and Clinical Studies: A Systematic Review of Evidence and Translational Barriers

**DOI:** 10.3390/biomedicines14040761

**Published:** 2026-03-26

**Authors:** Syed Haris Omar, Md Ahsan Ghani

**Affiliations:** 1 School of Dentistry and Medical Sciences, Faculty of Science and Health, Charles Sturt University, Orange, NSW 2800, Australia; 2 School of Agriculture, Environment and Veterinary, Charles Sturt University, Wagga Wagga, NSW 2650, Australia

**Keywords:** neurodegenerative diseases, Alzheimer’s disease, Parkinson’s disease, olive biophenols, oleuropein, hydroxytyrosol, oleocanthal

## Abstract

**Introduction:** Olives have been used in traditional Mediterranean medicine for thousands of years to address the causes of inflammation, ageing and cognitive health. Traditional preparations of olive include olive oil and olive leaf extract, which are major components of diets that contribute to maintaining cognitive function and reducing neurodegenerative disease risk. **Aims of the study:** This systematic review aimed to synthesise experimental and limited human evidence on olive biophenols in neurodegenerative disease models, identify the most studied compounds, characterise their mechanisms of action, and evaluate key translational barriers. **Materials and methods:** Following PRISMA 2020 guidelines and registered with PROSPERO (CRD420251252252), primary studies investigating the effects of well-characterised olive biophenols in neurodegenerative relevant in vitro, in vivo, or human models were systematically reviewed. Each study was assessed for its design, experimental model, mechanistic outcomes and reported limitations. Risk of bias was evaluated using validated tools (SYRCLE/OHAT/ToxR) appropriate for preclinical and experimental study designs. **Results:** Among the 25 studies, 7 (28.0%) examined oleuropein or oleuropein aglycone, 10 (40.0%) focused on hydroxytyrosol or its derivatives, and 9 (36.0%) investigated oleocanthal. Most studies employed in vivo animal models (57.7%), predominantly transgenic mouse models of AD and toxin-induced PD models. Oleuropein-based studies reported inhibition of amyloid-β and α-synuclein aggregation with behavioural improvements. Hydroxytyrosol primarily exerted antioxidant and anti-inflammatory effects with modest cognitive benefits. Oleocanthal showed the most consistent anti-amyloid and anti-tau activity, including enhanced amyloid-β clearance across the blood–brain barrier. Most studies show a moderate risk of bias due to incomplete reporting, randomisation and blinding. **Conclusions:** Olive biophenols demonstrate consistent neuroprotective effects in preclinical models; however, translation to clinical application remains limited by pharmacokinetic constraints, methodological heterogeneity, and insufficient human evidence.

## 1. Introduction

### 1.1. Neurodegenerative Disease Mechanisms

**Figure 1:**
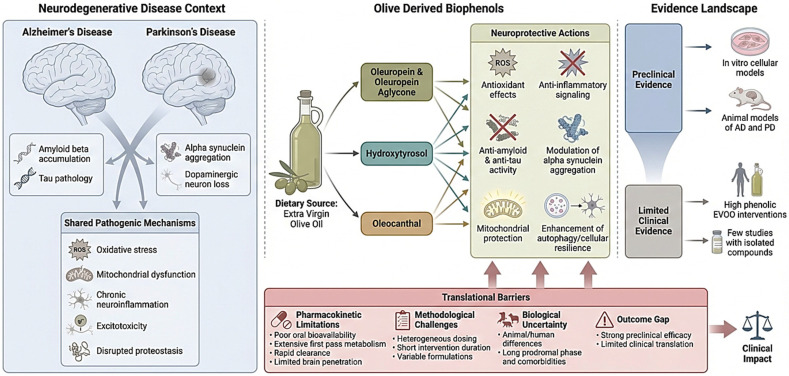
Conceptual overview of the neuroprotective potential and translational limitations of olive-derived biophenols in neurodegenerative diseases. The figure illustrates shared pathogenic mechanisms underlying Alzheimer’s disease and Parkinson’s disease, including oxidative stress, mitochondrial dysfunction, chronic neuroinflammation, excitotoxicity, and disrupted proteostasis. Olive-derived biophenols, primarily oleuropein and its aglycone, hydroxytyrosol, and oleocanthal, sourced from extra virgin olive oil, are shown to exert multimodal neuroprotective actions across experimental models, including antioxidant, anti-inflammatory, anti-amyloid, mitochondria-protective, and autophagy-enhancing effects. Despite strong preclinical evidence from in vitro and animal studies, clinical evidence remains limited and largely derived from high-phenolic extra virgin olive oil interventions. Key translational barriers highlighted include poor bioavailability, extensive metabolism, limited brain penetration, methodological heterogeneity, biological differences between models and human disease, and an outcome gap between preclinical efficacy and clinical translation.

Neurodegenerative diseases represent a diverse category of conditions that result in irreversible and progressive brain damage through the destruction of both the structure and function of brain cells (Figure 1). The two most well-known of these neurodegenerative diseases, Alzheimer’s disease (AD) and Parkinson’s disease (PD), have been shown to affect millions of people worldwide and impose significant economic burdens on society and on healthcare systems [1,2]. AD is primarily identified by the accumulation of amyloid beta (Aβ) peptides outside of cells and hyperphosphorylation of tau protein within cells [3]. PD is characterised by the degeneration of dopamine-producing cells in the substantia nigra and the abnormal aggregation of alpha-synuclein (αSN) [1,4].

Although the clinical manifestations of these neurodegenerative diseases differ significantly, they do share many common underlying pathologic processes. These include oxidative stress, mitochondrial dysfunction, chronic inflammation, excitotoxicity and disruptions of cellular proteostasis (Figure 1). As each of these processes continues to evolve and accumulate, the likelihood of cell death increases [1,5]. At present, there are few therapeutic options available to treat either of these diseases. Pharmacological treatments that are currently available for AD include cholinesterase inhibitors, and for PD include levodopa (L-DOPA). All of these drugs provide symptomatic relief, but neither drug can slow disease progression [1]. Thus, a major unmet medical need is to develop disease-modifying treatments for these neurodegenerative diseases that address multiple aspects of disease pathology while maintaining acceptable safety profiles. Parkinson’s disease models included toxin-induced dopaminergic degeneration models such as rotenone and 6-OHDA systems as well as models examining alpha-synuclein aggregation. These models provide mechanistic insight into the mitochondrial dysfunction, oxidative stress, and proteostasis disruption central to PD pathogenesis (Figure 1).

### 1.2. Olive Biophenols and Mediterranean Diet Evidence

In response to the limited success of conventional therapies, researchers have increasingly turned their attention to bioactive compounds found in food. In particular, researchers have focused on those foods commonly consumed as part of the traditional Mediterranean diet, which has long been associated with improved cognitive performance and decreased risk of developing neurodegenerative diseases [6,7,8,9]. One of the key components of the Mediterranean diet is extra virgin olive oil (EVOO) derived from *Olea europaea* L., which is known to be high in phenolic compounds often referred to as biophenols or polyphenols. These compounds are responsible for the flavour and aroma of EVOO and have been shown to exhibit a variety of beneficial biological effects (Figure 1) [6,7,8,10,11].

The plant olive (*Olea europaea* L.) has traditionally been used in medicine for centuries, and modern studies have provided evidence of both the neuroprotective and anti-ageing effects of olive biophenols or polyphenols (oleuropein, hydroxytyrosol, oleocanthal, etc.), which have contributed to the preservation of cognitive function in ageing individuals [12,13]. Ethnobotanical surveys and ethnopharmacological documentation from the Mediterranean region, the Middle East and Africa demonstrate a wide variety of uses for various parts of olive (oil, leaves, bark, and fruit) to treat symptoms of ageing and those related to the nervous system, including inflammation, pain, fever, arthritis, cardiovascular diseases, hypertension and diabetes complications [12,14,15]. In addition, decoctions and infusions of the leaves and fruits have been reported for “nerve” and brain-related ailments, such as diabetic neuropathic pain and stroke in laboratory models, suggesting that some of the traditional indications may be valid.

As an example, olive leaf tea and tinctures are commonly used as blood pressure-lowering and anti-inflammatory agents in Greece, Italy, Morocco, Palestine, Algeria, and East Africa, which can help to alleviate some of the vascular and inflammatory contributors to cognitive decline and stroke [12]. Evidence in modern studies relating to neurological and age-related health studies on olive biophenols or polyphenols (oleuropein, hydroxytyrosol, oleuropein aglycone, and oleocanthal) has demonstrated neuroprotection and cognitive support: antioxidant, anti-inflammatory, anti-amyloidogenic, anti-tau, neuroprotective in animal models of AD, PD, stroke, multiple sclerosis, depression and anxiety [16]. The consumption of EVOO, particularly high-phenolic EVOO, has shown improvement in cognition or slowed cognitive decline in older adults and in patients with mild cognitive impairment and has been found to be inversely associated with the risk of developing AD and other dementias [17]. In addition, olive biophenols modulate Nrf2, AMPK, autophagy and proteostasis to promote healthy ageing, extend lifespan in animal models and attenuate sarcopenia [18].

### 1.3. Major Olive Biophenol Compounds

Among the many phenolic constituents identified in olive products, oleuropein (OLE), hydroxytyrosol (HT) and oleocanthal (OC) are some of the most well-studied biophenols isolated from olive (*Olea europaea* L.) products and have been shown to possess antioxidant, anti-inflammatory and anti-amyloidogenic activity. Collectively, these data support the potential of these compounds as protective agents against neurodegeneration [6,8,10,19,20]. Preclinical investigations have shown that these compounds can interfere with pathogenic protein aggregation, attenuate oxidative and inflammatory damage, preserve mitochondrial function, and modulate intracellular signalling pathways implicated in neurodegeneration [6,21,22]. Beyond classical antioxidant effects, emerging evidence suggests that olive biophenols may influence gene expression and neuronal resilience through epigenetic and transcriptional mechanisms, further expanding their therapeutic potential (Figure 1) [6].

### 1.4. Translational Challenges and Objectives

Despite a growing body of experimental research, the translation of olive biophenols from bench to bedside remains limited. Variability in study design, differences in compound formulation and dosage, limited bioavailability, and a scarcity of well-designed clinical trials pose significant challenges to clinical implementation. A comprehensive synthesis of the available evidence is therefore essential to clarify the current state of knowledge, identify the most promising compounds, and delineate the barriers hindering their therapeutic translation.

Several studies examined the association between the consumption of EVOO and a reduced risk of neurodegenerative diseases [17,23,24]; however, most studies have focused on EVOO within a composite dietary framework or alongside biophenols or polyphenol extracts. Therefore, based upon the evidence in these studies, it is difficult to determine what role each individual bioactive compound may play in the reported neuroprotection. Additionally, the composition of EVOO from *Olea europaea* can vary significantly depending upon the type of olive cultivar used, how the oil was processed and stored, and the amount consumed. As a result, there is significant variation in the concentration and types of biophenols present in different oils and the resultant biological responses. Finally, studies that use whole-extract approaches have complicated pharmacokinetic interpretations, dose standardisation, and regulatory translation.

While the evaluation in this review will focus on biophenol (bioactive compound) individually, it is understood that an examination of each biophenol individually does not indicate that isolated bioactive compounds produce the same effect as the preparation that has been historically used. Therefore, studies examining single compounds should be viewed as mechanistic tools for understanding how specific components of a bioactive matrix contribute to overall biological function. When applicable, the comparison of whole extracts vs. purified compounds will also be highlighted to evaluate if the demonstrated biological effect can be attributed to one compound or to the interactions of the multiple phenolic compounds found in olive.

Therefore, this highlights the importance of examining olive biophenols as discrete pharmacologic agents to establish compound-specific mechanisms of action, to establish the range of doses at which these compounds can be safely consumed, and to evaluate the translatability of findings from preclinical models to humans. The evaluation of single compounds also provides an opportunity to critically compare results obtained using whole-extract and multi-compound approaches and to determine if the neuroprotective effects associated with EVOO consumption are due to the activity of specific compounds, to synergies among the compounds in the extract, or to some other aspect of the EVOO matrix.

Accordingly, this systematic review aims to critically evaluate the existing evidence on olive-derived biophenols in neurodegenerative diseases in order to (i) identify the most frequently investigated olive biophenols, (ii) characterise the neuroprotective mechanisms attributed to these compounds, (iii) assess the strength and consistency of evidence supporting their efficacy across preclinical and clinical studies, and (iv) examine the key translational barriers, with particular emphasis on pharmacokinetic limitations, methodological heterogeneity, and unresolved biological questions. Collectively, these objectives address the central question of this review: Which olive-derived biophenol components have been most extensively studied in neurodegenerative disease models and clinical settings, how robust is the evidence supporting their neuroprotective effects, and what critical factors currently limit their clinical translation? (Figure 1).

The goal of this study is not simply to record the neuroprotective results of olive-derived biophenolic compounds; however, it will seek to understand why promising research has failed to be translated into successful therapeutic application for patients with AD and PD by identifying the pharmacokinetics and models, dose feasibility, and gaps in evidence which are the defining elements of the translational barrier for this class of bioactive compounds.

## 2. Methods

**Figure 2:**
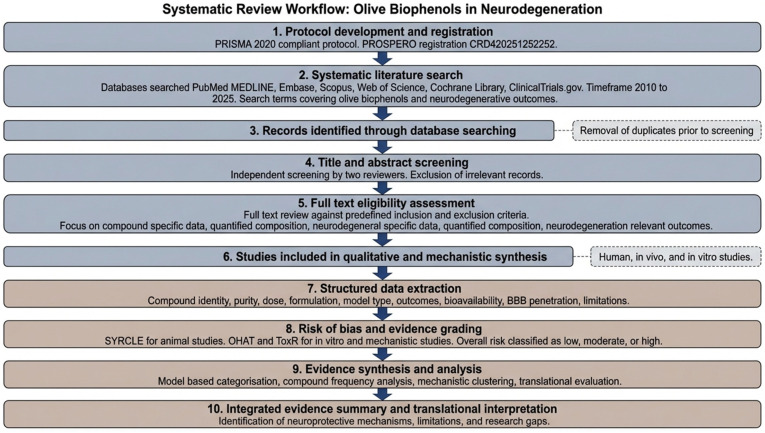
Overview of the systematic review methodology, including protocol development and registration, literature search, study selection, data extraction, risk of bias assessment, and evidence synthesis.

This systematic review was performed following the Preferred Reporting Items for Systematic Reviews and Meta-Analyses (PRISMA) guidelines [25]. Under registration number CRD420251252252, the protocol, inclusion criteria, and analysis methods were registered with the Prospective Register of Systematic Reviews (PROSPERO—http://www.crd.york.ac.uk/PROSPERO/, accessed on 11 February 2026) (Figure 2).

### 2.1. Search Strategy and Data Sources

A systematic search for relevant literature was conducted across several electronic databases (Figure 2). These included PubMed/MEDLINE, Embase, Scopus, Web of Science Core Collection, Cochrane Library, and ClinicalTrials.gov. The search encompassed studies published within the last 15 years (2010–2025) to capture recent advancements in this rapidly developing field. The search strings combined the following terms: (“olive” OR “olea europaea” OR “olive oil” OR “Extra Virgin Olive oil” OR “olive leaf”) AND (“polyphenol*” OR “biophenol*” OR “hydroxytyrosol” OR “oleuropein” OR “oleocanthal” OR “tyrosol” OR “oleacein” OR “verbascoside”) AND (“neurodegenerative” OR “Alzheimer*” OR “Parkinson*” OR “dementia” OR “cognitive” OR “neuroprotection” OR “brain” OR “neuron*”) AND (“human” OR “clinical” OR “trial” OR “animal” OR “mouse” OR “rat” OR “in vitro” OR “cell”).

### 2.2. Inclusion and Exclusion Criteria

Studies were included if they investigated well-characterised olive biophenols, including hydroxytyrosol, oleuropein, oleocanthal, tyrosol, verbascoside, or ligstroside, or defined olive extracts with quantified phenolic composition (Figure 2). Eligible studies examined neurodegeneration-relevant outcomes in human clinical trials, in vivo animal models, or in vitro neuronal or glial systems. Studies lacking compositional data, unrelated to neurodegeneration or brain health, unavailable in full text, published in languages other than English, or classified as reviews, editorials, protocols, or guidelines were excluded. Studies using supraphysiological concentrations were critically scrutinised.

### 2.3. Screening Procedures

Title and abstract screening were done independently by two reviewers. Full-text assessment and data extraction were performed independently by two reviewers (S.H.O. and M.A.G.), with discrepancies resolved through discussion and, where necessary, adjudication by a third independent reviewer. This approach aligns with recommended methodological standards for systematic reviews and was implemented to minimise selection and extraction bias. The screening process was carried out in two phases as depicted in Figure 2. In phase 1, titles and abstracts retrieved through the database search were screened for relevance to olive polyphenols and neurodegenerative research. In phase 2, full-text versions of potentially eligible studies were assessed against predefined criteria for inclusion or exclusion, with emphasis on studies that examined individual compounds, reported quantitative composition of extracts, and focused on neurodegeneration related outcomes. Duplicate records were removed before the first screening phase was initiated.

### 2.4. Data Extraction

Information from the selected articles was systematically extracted using a pre-designed data sheet (Figure 2). Key data points included the specific olive biophenol studied, their purity, dose, and formulation, the study model (human trial type, animal model, or cell type), and primary outcomes with reported effect sizes. Additionally, the data extraction captured details on bioavailability, blood–brain barrier (BBB) penetration if reported, and any explicit or implicit limitations noted by the authors or identified during the review (e.g., lack of dose–response data, small sample sizes). This structured approach facilitated comprehensive data capture for subsequent analysis.

### 2.5. Risk of Bias Assessment and Evidence Grading

Risk of bias was assessed for all included studies in accordance with the PRISMA 2020 recommendations, using tools appropriate for each study design. In light of the significant number of preclinical studies as well as the different types of study models, several tools were used to assess the risk of bias in accordance with the type of experiment performed. The Cochrane-based Systematic Review Centre for Laboratory Animal Experimentation (SYRCLE) risk of bias (RoB) tool [26] was used to evaluate the quality of the animal in vivo studies by assessing six elements, including randomisation, allocation concealment, blinding, completeness of all recorded outcomes, selective reporting, and other potential biases. For in vitro and mechanistic studies, the National Toxicology Program’s M-OHAT (Modified Office for Health Assessment and Translation) and ToxR (Toxicological Data Reliability) assessment tool framework [27] were utilised to assess three components of study design, including the characterisation of exposures, the use of appropriate controls, and the reporting of results; the need for randomisation or blinding was considered to be inappropriate if it did not apply to the research design (Figure 2). Studies utilising the nematode worm *Caenorhabditis elegans* were evaluated using a version of the SYRCLE methodology that accounted for the specific limitations inherent to this model system. Each domain was rated as low risk, some concerns, high risk, or unclear risk, and an overall risk of bias judgement (low, moderate, or high) was assigned based on the highest level of concern. The evidence strength classification criteria used included four categories: the number of independent studies that reported similar results, replication of the results across the various experimental models, relevance of the data collected to the neurodegenerative pathological processes being studied, and methodological quality of the study as determined by risk of bias assessment. Strong evidence would include replication of the results in multiple in vivo models of disease, with mechanistic support. Moderate evidence would consist of support from multiple preclinical studies, although no replication occurred across models. Limited evidence would be defined as evidence from in vitro studies or evidence from only one model.

Acute biochemical assays, usually conducted in vitro over hours to days, served as mechanistic indicators of potential neuroprotection. In contrast, chronic interventions, extending for weeks or months in vivo within animal models, were employed to assess the translational feasibility of enduring neuroprotective effects. Although biochemical assays demonstrate that these compounds (oleuropein and oleocanthal) can inhibit protein aggregation in vitro, this does not necessarily translate into sustained neuroprotective effects in long-term in vivo disease models.

Furthermore, due to the significant variability in experimental design across studies, the weighting of each study in relation to the overall evidence strength considered not only the quantity of studies reporting positive results but also the consistency of mechanistic outcomes across diverse experimental systems.

### 2.6. Evidence Synthesis and Analysis

The included studies were systematically analysed and categorised according to the experimental model (in vivo animal models, in vitro cellular or biochemical systems, and human studies). Given that the evidence base consisted predominantly of preclinical investigations, the quality and interpretability of evidence were assessed by considering the study design characteristics, internal consistency of findings, relevance of outcome measures to neurodegenerative pathology, and translational plausibility. A compound frequency analysis was conducted to identify the olive biophenols most extensively investigated. Mechanistic synthesis was performed by clustering reported outcomes into predefined biological domains, including anti-amyloid and anti-tau actions, antioxidant and anti-inflammatory effects, mitochondrial protection, autophagy modulation, and signalling or epigenetic regulation. Where reported, dose ranges, routes of administration, and treatment duration were extracted and qualitatively compared across studies; formal dose–response modelling was not undertaken due to methodological heterogeneity. Translational considerations were evaluated by examining the reported pharmacokinetic information, the feasibility of achieving effective concentrations in vivo, and the biological relevance of the experimental models. Identified limitations were systematically categorised as pharmacokinetic, methodological, or biological/translational barriers (Figure 2).

## 3. Results

### 3.1. Identification of Studies

**Figure 3:**
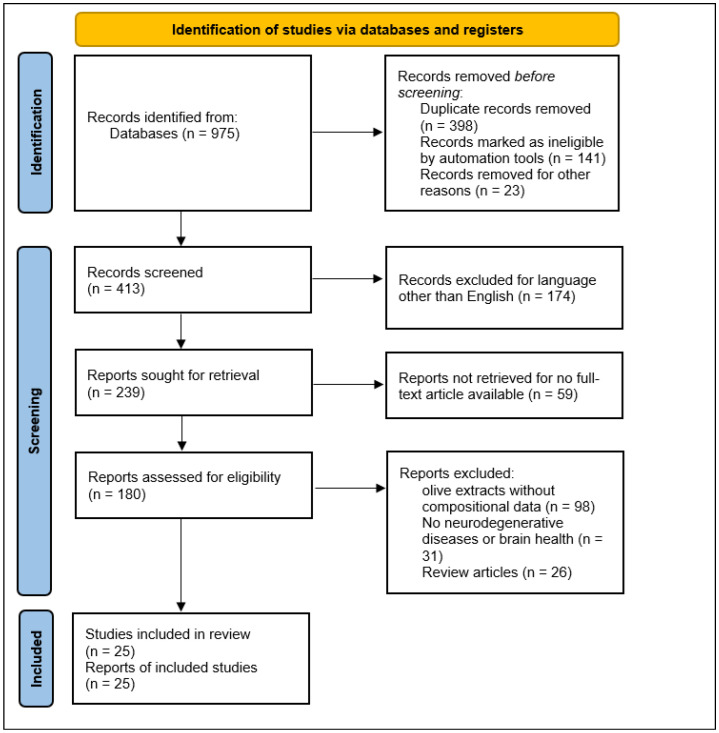
PRISMA 2020 flow diagram illustrating the identification, screening, eligibility assessment, and inclusion of studies in the systematic review.

Out of 975 records identified, 413 were screened after the removal of duplicates and ineligible records. Following title/abstract screening, full-text assessment of 180 reports resulted in 25 studies being included in the review, with exclusions mainly due to a lack of compositional data, irrelevant outcomes, or review-type publications (Figure 3).

### 3.2. Characteristics of the Studies

Table 1 presents an overview of the characteristics of all studies included in the systematic review. In total, 25 unique experimental studies were included in the systematic review. However, there are 26 component-specific studies included because one study evaluated two olive biophenols simultaneously [28]. Of those studies included within the review, seven (28.0%), ten (40.0%), and nine (36.0%), respectively, assessed either oleuropein or oleuropein aglycone; hydroxytyrosol or its derivatives; and oleocanthal or a combination of the two biophenols. As several studies evaluated more than one biophenol, the total number of component-specific analyses exceeds the number of included studies.

The majority of studies employed in vivo experimental models (15/25, 60.0%), including transgenic mouse models of AD pathology (e.g., TgCRND8, APP/PS1, TgSwDI, and 5xFAD), toxin-induced Parkinson’s disease models, and Caenorhabditis elegans models of neurodegeneration and ageing. The remaining 10/25 studies (40.0%) used in vitro cellular or biochemical systems, primarily addressing oxidative stress, mitochondrial dysfunction, neuroinflammation, or protein aggregation processes. From a disease-context perspective, AD-related models predominated, particularly among oleocanthal and oleuropein aglycone studies, which frequently examined Aβ aggregation, tau fibrillisation, and amyloid clearance mechanisms. PD models represented the second most common disease context, especially in studies of OLE and HT, focusing on dopaminergic neuroprotection, α-synuclein aggregation, mitochondrial impairment, and autophagy. HT studies additionally explored mitochondrial bioenergetics, oxidative stress, neuroinflammation, and cognitive or behavioural outcomes across multiple experimental systems.

Most of the studies found a moderate amount of bias based on a major lack of reporting on randomisation and blinding; however, many of the mechanisms for the findings were reported consistently by different labs using independent models. While the findings from these studies are biologically plausible, they are limited by a lack of methodological clarity, which limits confidence in their ability to replicate and measure the size of the effect. The neuroprotective benefits discussed in this paper should therefore be viewed as plausibly biologic but not clinically proven.

Overall, while the overall results from each study indicated a positive finding for neuroprotection, based on the methodological quality of the studies included in the review, most studies were determined to be at a moderate risk of bias. The primary reason for determining the studies to have a moderate risk of bias was due to the lack of complete reporting of methods used for randomisation, allocation concealment, and blinding, rather than an issue with the methods used for assessing outcomes or issues with selective reporting.

### 3.3. Characteristics of Olive Biophenol Components

This systematic review comprised studies of OLE or oleuropein aglycone (7/25, 28.0%), HT and its derivatives (10/25, 40.0%), and oleocanthal (9/25, 36.0%) (Table 2).

With respect to experimental design, 15 studies (15/25, 60.0%) employed in vivo animal models, including transgenic mouse models of AD, toxin-induced Parkinson’s disease models, stress-based behavioural paradigms, and *Caenorhabditis elegans*, whereas 10 studies (10/25, 40.0%) were conducted using in vitro or biochemical systems, such as neuronal or glial cell lines, blood–brain barrier models, and protein aggregation assays.

Regarding exposure characteristics, oral administration was used in 15 studies (15/25, 60.0%), primarily through dietary supplementation or oral gavage in animal models, while direct in vitro exposure accounted for 10 studies (10/25, 40.0%). Treatment duration closely mirrored study design: acute exposure protocols (hours to days) were applied in 10 studies (10/25, 40.0%), corresponding to in vitro investigations, whereas longer-term interventions lasting weeks to months were employed in 15 studies (15/25, 60.0%), all of which were in vivo.

Across components, outcome measures were mechanistically aligned with disease-relevant pathways. Oleuropein-based studies predominantly assessed Aβ or α-synuclein aggregation, mitochondrial dysfunction, autophagy, and cognitive or motor outcomes. HT studies primarily focused on oxidative stress markers, mitochondrial bioenergetics, neuroinflammatory signalling, neurotrophic pathways, and behavioural or cognitive performance. OC investigations mainly evaluated Aβ clearance, tau fibrillisation, neuroinflammation, blood–brain barrier transport, and cognitive or metabolic phenotypes. Despite heterogeneity in dosing and exposure duration, endpoint selection was consistently directed towards the core pathological mechanisms underlying neurodegenerative disease.

### 3.4. Analysis Approach (Strength and Consistency of Evidence)

Across the 26 component-specific investigations, the strength and consistency of evidence varied by biophenol and experimental context. Oleuropein and oleuropein aglycone were supported by moderate-to-strong preclinical evidence, with convergent findings reported across cellular, invertebrate, and mammalian models, including transgenic AD and toxin-induced Parkinson’s disease models [32,36,38,45]. HT and its derivatives demonstrated moderate and generally consistent evidence, particularly for antioxidant, anti-inflammatory, and mitochondrial-protective effects, although several studies relied on indirect or non-disease-specific models [40,42,50,51]. Oleocanthal showed the strongest mechanistic consistency, with reproducible effects across biochemical, cellular, and transgenic mouse models of AD [29,30,37,44,47]. Nevertheless, evidence across all components remains predominantly preclinical, with no controlled clinical trials evaluating isolated compounds (Table 3).

### 3.5. Key Findings

**Figure 4:**
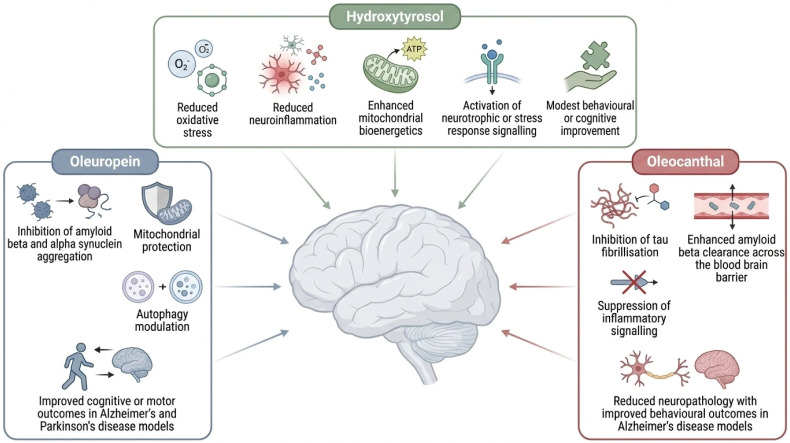
The complementary and partially overlapping neuroprotective actions of key olive-derived biophenols in preclinical models of Alzheimer’s and Parkinson’s diseases. Oleuropein primarily targets protein aggregation, mitochondrial protection, and autophagy, leading to improved cognitive and motor outcomes. Hydroxytyrosol predominantly modulates oxidative stress, neuroinflammation, mitochondrial bioenergetics, and stress response signalling, with modest behavioural benefits. Oleocanthal shows strong anti-amyloid and anti-tau activity, enhances amyloid beta clearance across the blood–brain barrier, suppresses inflammatory signalling, and improves neuropathological and behavioural outcomes, highlighting mechanistic complementarity across compounds.

Collectively, the included studies indicate that olive-derived biophenols target multiple core pathological mechanisms implicated in neurodegeneration. Oleuropein-based interventions were most consistently associated with inhibition of Aβ and α-synuclein aggregation, preservation of mitochondrial function, modulation of autophagy, and improvements in cognitive or motor outcomes in Alzheimer’s and Parkinson’s disease models [31,36,38,45]. HT studies predominantly reported reductions in oxidative stress and neuroinflammation, enhancement of mitochondrial bioenergetics, activation of neurotrophic or stress-response signalling pathways, and modest improvements in behavioural or cognitive performance [40,42,43,48,51]. Oleocanthal investigations consistently demonstrated direct inhibition of tau fibrillisation, enhanced Aβ clearance across the BBB, suppression of inflammatory signalling, and attenuation of neuropathological and behavioural deficits in AD models [30,33,37,44,47] (Figure 4 and Table 3).

### 3.6. Key Translational Barriers

**Figure 5:**
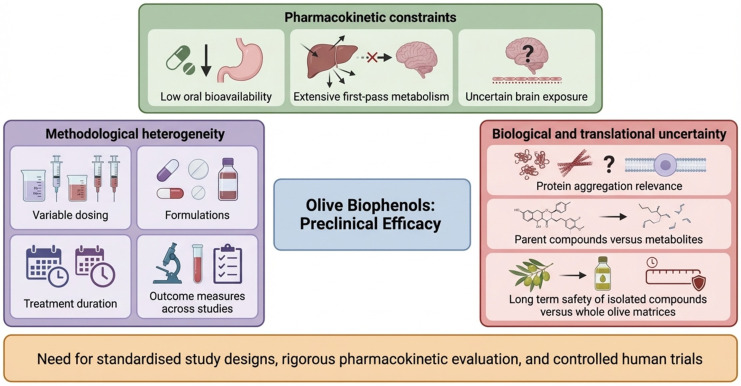
The major translational barriers that limit the progression of olive-derived biophenols from promising preclinical findings to effective clinical interventions. Key challenges include pharmacokinetic constraints such as low oral bioavailability, extensive first-pass metabolism, and uncertain brain exposure. Methodological heterogeneity across studies, including variable dosing, formulations, treatment duration, and outcome measures, further complicates evidence interpretation. Biological and translational uncertainties, particularly the relevance of protein aggregation targets, the activity of metabolites versus parent compounds, and the long-term safety of isolated biophenols compared with whole-olive matrices, collectively highlight the need for standardised study designs, rigorous pharmacokinetic evaluation, and well-controlled human trials.

Despite encouraging preclinical findings, several shared translational barriers limit clinical advancement of olive biophenols. A major limitation across compounds is pharmacokinetic constraints, including low oral bioavailability, extensive first-pass metabolism, and uncertain brain exposure, which complicates extrapolation from effective experimental doses to human application (Figure 5) [30,44,45]. Methodological heterogeneity further limits interpretability, with wide variability in dosing strategies, formulations, treatment duration, and outcome measures across studies (Figure 5) [36,41,47]. In addition, several unresolved biological questions remain, including the relevance of protein aggregation assays to disease modification [37,39], the contribution of metabolites versus parent compounds [43,51], and the long-term safety and efficacy of isolated compounds compared with whole-olive matrices (Figure 5) [29,41]. These limitations highlight the need for standardised designs, rigorous pharmacokinetic studies, and controlled human trials (Figure 5 and Table 3).

## 4. Discussion

Although OLE, HT, and OC are frequently referenced in conjunction with each other as a result of their shared dietary source (i.e., olive oil), they are chemically distinct, metabolised differently, exhibit varying pharmacokinetic profiles, and appear to exert different mechanisms of action [53,54]. Therefore, this review evaluates compound-specific evidence without assuming equivalence among the aforementioned compounds or implying synergy when interpreting composite effects generated from the administration of whole-EVOO or extracts. Rather, whole-EVOO and extract-based findings are viewed as composite effects that may reflect either additive or synergistic interactions that cannot be replicated through the use of individual molecules. The outcome of the systematic review demonstrates that research on olive-derived biophenols in neurodegenerative diseases is highly concentrated on three compounds, OLE (and oleuropein aglycone), HT, and OC, which together account for all component-specific investigations included in this review. This pattern aligns with the broader literature indicating that these compounds are the most abundant and biologically active phenolics in olives and EVOO [8,10,55]. Other phenolics such as tyrosol, verbascoside, and ligstroside are less frequently investigated as isolated agents and are typically studied within complex extracts or whole-olive products [56,57].

The literature screening was also used to identify a variety of additional olive phenols, such as verbascoside, ligstroside, oleacein and tyrosol. These compounds were found to be studied largely as part of larger mixtures of olive phenols and as part of dietary intervention, but they have rarely been studied as individual compounds in models of neurodegeneration. Consequently, fewer than three studies were identified that evaluated one of these compounds as an individual compound with mechanistic outcomes that are relevant to diseases. For this reason, the present review will focus on the three phenolic compounds most extensively studied as single compounds, i.e., oleuropein (and its aglycone) and oleocanthal and hydroxytyrosol.

In addition, the fact that olive oil and leaf preparations have been traditionally consumed by people in many regions around the world for centuries provides support for the view that these compounds are ethnopharmacologically relevant agents. Additionally, it is important to consider how the traditional consumption of whole plant material relates to the results of studies on isolated compounds.

Consistent with prior reviews [6,10], the evidence base is predominantly preclinical, with the majority of studies employing in vivo animal models of AD and PD, followed by in vitro cellular and biochemical systems. Human evidence remains limited and is largely derived from interventions using high-phenolic EVOO rather than isolated biophenols [58]. Across the included studies, AD-related models were most frequently investigated, particularly in oleuropein aglycone and OC studies, which focused on Aβ aggregation, tau fibrillisation, and amyloid clearance mechanisms. PD models were the second most common, especially for OLE and HT, where outcomes related to dopaminergic neuron survival, α-synuclein aggregation, oxidative stress, and mitochondrial dysfunction predominated [1,6].

Mechanistically, the findings across Table 2 and Table 3 indicate that olive biophenols exert multimodal neuroprotective actions, rather than acting through a single dominant pathway. Anti-amyloid and anti-tau effects were particularly evident for oleuropein aglycone and OC, while HT more consistently targeted oxidative stress, mitochondrial bioenergetics, and neuroinflammatory pathways [10,59]. These complementary mechanisms support the hypothesis that olive biophenols may be most effective in early or preventive contexts, where modulation of multiple pathological processes may slow disease progression rather than reverse established neurodegeneration.

OLE, a glycosylated secoiridoid abundant in olive leaves and fruits, and its aglycone derivative emerge as key neuroprotective agents. The included studies demonstrate that oleuropein aglycone consistently reduces Aβ plaque burden, mitigates synaptic dysfunction, and improves cognitive or motor outcomes in AD and PD models [6,60]. Importantly, oleuropein aglycone shows direct interference with amyloidogenic protein aggregation, including both Aβ and α-synuclein, redirecting aggregation pathways towards less toxic species [61]. Despite robust preclinical efficacy, clinical translation remains limited, largely due to oleuropein’s poor oral bioavailability and extensive metabolic conversion [55,62]. Most human studies rely on olive oil or leaf extracts containing multiple phenolics, making it difficult to attribute the observed effects specifically to oleuropein [6].

HT, a phenylethanoid derived from oleuropein hydrolysis, exhibits a distinct neuroprotective profile, characterised by strong antioxidant, anti-inflammatory, and mitochondria-supportive actions [8,10]. Compared with OLE, HT demonstrates superior oral bioavailability, with measurable plasma levels following dietary intake [55,57]. In animal models, HT improves cognitive performance, reduces oxidative damage, and attenuates neuroinflammation across AD- and PD-relevant systems [6]. However, its direct anti-amyloid effects appear less pronounced than those of OC or oleuropein aglycone [59]. Human studies using high-phenolic EVOO suggest cognitive benefits, but the specific contribution of HT remains difficult to isolate due to the complex phenolic matrix of olive oil [58]. Only three human studies have examined purified HT in isolation to date; thus, the majority of the clinical evidence available in support of the anti-inflammatory, cardioprotective, and neuroprotective effects of phenolic-rich EVOO has been obtained from studies examining the effects of high-phenolic EVOO consumption rather than those examining the effects of administering single compounds [63,64,65].

OC is distinguished by its unique aldehyde structure, conferring potent anti-inflammatory and anti-amyloid properties [10]. Mechanistically, OC directly inhibits Aβ oligomerisation and fibrillisation, promotes amyloid clearance, and modulates tau aggregation, positioning it as a particularly promising candidate for AD-related pathology [6]. Preclinical evidence from both cellular and animal models consistently demonstrates a reduced amyloid burden and improved behavioural outcomes following OC administration. However, as with OLE, human evidence for isolated OC is scarce, and challenges related to bioavailability, stability, and BBB penetration remain significant translational barriers [62].

Olive biophenols share mechanistic overlap with other plant-derived compounds such as flavonoids, lignans, and triterpenoids, which also target oxidative stress, inflammation, and protein aggregation [4,66,67]. However, olive biophenols are distinguished by direct interactions with amyloidogenic proteins, particularly Aβ and α-synuclein, and by epidemiological support from Mediterranean diet studies [6,10]. As with other natural compounds, limited brain bioavailability remains the primary obstacle to clinical efficacy [1,68].

Despite the robust and largely consistent neuroprotective effects observed across cellular and animal models, the translation of olive biophenols into clinically effective interventions for neurodegenerative diseases remains constrained by several interrelated barriers.

A major translational limitation lies in pharmacokinetic mismatch between experimental conditions and human physiology. Many in vitro studies employ micromolar concentrations of OLE, HT, or OC that are far above those achievable in human plasma or brain tissue following oral intake [62]. Even in animal studies, administered doses often exceed realistic dietary or supplement-equivalent exposures, limiting direct extrapolation to human use. This discrepancy contributes to the recurrent gap between promising preclinical efficacy and modest or inconsistent clinical outcomes.

Mechanisms for signalling from antioxidants (such as Nrf2 and mitochondria) are likely to exist at levels low enough to be reached through diet, whereas inhibition of protein aggregation in in vitro systems often requires concentrations of the compound greater than those found in the body after typical doses. As a result, while there is some basis to consider inhibiting the formation of protein aggregates as a possible mechanism of action, it should not currently be considered a confirmed therapeutic activity in vivo.

Extensive metabolism and low oral bioavailability further complicate translation. Olive biophenols undergo rapid phase I and phase II metabolism in the gastrointestinal tract and liver, resulting in circulating metabolites that differ substantially from the parent compounds tested in vitro [55,57]. While HT demonstrates comparatively better absorption than OLE or ligstroside, it is also rapidly conjugated and eliminated, reducing sustained systemic exposure [55]. Importantly, the biological activity of these conjugated metabolites within the central nervous system remains insufficiently characterised, introducing uncertainty regarding their true therapeutic relevance. The gut microbiome may act as an important metabolic organ influencing the biotransformation of olive biophenols. Microbial metabolism can convert oleuropein into hydroxytyrosol and further into phenolic acids that may themselves possess biological activity. Consequently, the neuroprotective effects observed following oral administration may reflect a combined influence of parent compounds, circulating metabolites, and microbiota-derived phenolic derivatives. An unresolved question concerns whether glucuronidated or sulfated metabolites represent inactive excretion products or function as circulating reservoirs that can be locally deconjugated at target tissues. Enzymes such as β glucuronidase, which are often elevated in inflamed tissues, may regenerate active parent compounds locally. Investigation of this prodrug-like mechanism represents an important future research direction.

In the future, translational studies attempting to elucidate olive biophenols metabolism and central bioavailability, pharmacokinetics and metabolomics studies should be combined. High-resolution liquid chromatography coupled to mass spectrometry will be able to accurately quantify parent biophenols and their metabolites in blood plasma, cerebrospinal fluid and brain homogenates. Further studies employing brain microdialysis and tracer studies with isotopically labelled derivatives may also contribute to determining whether circulating conjugated metabolites maintain some biological activity exerted via their parent compounds in the brain or whether they just serve as a repository from which the active compound is deconjugated to exert localised pharmacological effects within the brain. These studies are of paramount importance to establish whether the presence of biophenols and their metabolites in the CNS is relevant in terms of biological effects associated with active metabolites, and in reducing or preventing neurodegeneration due to the action of the parent compound, the biologically relevant metabolites in circulation or the products derived from microbiota catabolism.

A central limitation in the existing body of evidence is a reliance upon laboratory experiments using physiological conditions that do not mimic human physiology. Most in vitro studies use micromolar concentrations that are not likely to occur in human plasma or brain tissue; most in vivo studies use high doses that may exaggerate efficacy due to the short duration of treatment for chronic slow-progressing neurological disorders. At the same time, transgenic and toxin-based models represent many of the pathological features of an individual case of human disease but cannot capture the variability of clinical presentations, the co-morbidities associated with this group of disorders and the prodromal phase that can lasts years prior to diagnosis. Collectively, these constraints will elevate the likelihood of over-interpretation when translating the results of preclinical studies into clinical settings.

Another critical translational barrier is limited BBB penetration. Most olive biophenols, particularly glycosylated forms and polar metabolites, exhibit poor BBB permeability [1,66]. Consequently, even when systemic absorption occurs, brain concentrations may remain below thresholds required to replicate preclinical effects observed in vitro. This limitation likely explains why compounds such as oleocanthal and oleuropein aglycone, despite strong anti-amyloid activity in experimental systems, have yet to demonstrate clear efficacy in human trials [6,62].

Future clinical trials employing isolated olive phenolic compounds will require pharmacokinetic directed dosing strategies, including determination of plasma and cerebrospinal fluid levels of both the parent compounds and their metabolites. Micro-dosing studies and phase I pharmacokinetic studies can provide insight as to whether adequate brain exposure is possible. Additionally, the use of nanotechnology-based formulations, lipid-based drug delivery systems or prodrug derivatives may aid in enhancing the penetration of isolated biophenols through the blood–brain barrier and potentially improving pharmacokinetic stability.

From a methodological standpoint, heterogeneity in experimental design poses a significant challenge. Studies vary widely in compound source (synthetic vs. plant-derived), purity, formulation, route of administration, dose, and treatment duration. Moreover, olive biophenol preparations are often insufficiently characterised, with incomplete reporting of individual phenolic content, making cross-study comparisons and replication difficult [56]. This lack of standardisation limits the ability to define clear dose–response relationships or establish comparative efficacy across compounds.

Model validity represents another methodological concern. While animal models recapitulate selected pathological features of Alzheimer’s and Parkinson’s diseases, they do not fully reflect the complexity, heterogeneity, and long prodromal phases of human neurodegeneration [6]. Consequently, therapeutic effects observed in young or genetically homogeneous animal models may overestimate efficacy in aged, comorbid human populations. In addition, many studies use relatively short intervention periods, which may be insufficient to model chronic disease modification in slowly progressive disorders.

The moderate risk of bias identified across many studies reflects broader challenges in preclinical neuroscience research. Poor reporting of randomisation, allocation concealment, and blinding has been recognised as a contributor to the reproducibility crisis in experimental biomedical science. Adoption of reporting frameworks such as the ARRIVE guidelines may substantially improve methodological transparency and reliability of preclinical findings. Future systematic reviews may consider incorporating ARRIVE compliance criteria when evaluating evidence quality.

Finally, biological and translational uncertainties remain regarding synergistic effects, inter-individual variability, and long-term safety. Evidence increasingly suggests that the neuroprotective benefits of olive products may arise from the combined action of multiple phenolics within the food matrix rather than from isolated compounds alone [10]. Furthermore, genetic background, gut microbiota composition, age, and disease stage can markedly influence biophenol metabolism and response, contributing to variable outcomes across individuals [58,68]. Although olive biophenols are generally regarded as safe at dietary levels, long-term safety data for high-dose, isolated formulations in neurodegenerative populations are lacking, underscoring the need for rigorous toxicological and longitudinal studies.

Future studies will benefit from employing factorial experimental designs and systems biology approaches to investigate potential synergistic interactions among olive phenolics. Future studies will utilise combinatorial dosing studies and network pharmacology approaches to assess whether individual compounds additively or synergistically contribute to biological activity. Utilising metabolomics and transcriptomics will also facilitate understanding how mixtures of compounds interact with cellular signalling pathways, as compared to individual compounds.

Collectively, these translational and methodological limitations highlight the necessity for standardised compound characterisation, physiologically relevant dosing, advanced delivery strategies, and well-designed clinical trials to determine whether the promising preclinical neuroprotective properties of olive biophenols can be effectively harnessed in human neurodegenerative disease prevention or treatment.

## 5. Conclusions

**Figure 6:**
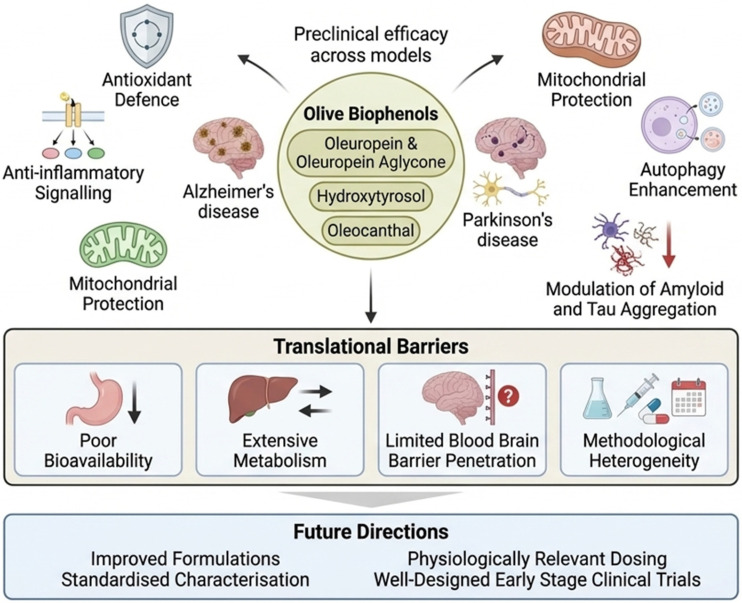
An integrated overview of the neuroprotective potential of olive-derived biophenols alongside the key challenges limiting their clinical translation.

Olive-derived biophenols, particularly oleuropein (and its aglycone), hydroxytyrosol, and oleocanthal exhibit reproducible neuroprotective effects across in vitro and animal models of Alzheimer’s and Parkinson’s diseases, while evidence in humans remains limited and largely indirect (Figure 6). These compounds act through complementary mechanisms involving antioxidant defence, anti-inflammatory signalling, mitochondrial protection, autophagy enhancement, and direct modulation of amyloidogenic protein aggregation (Figure 6). Despite strong preclinical support, clinical translation remains limited, primarily due to poor bioavailability, extensive metabolism, limited BBB penetration, and methodological heterogeneity (Figure 6). Current human evidence, largely derived from high-phenolic EVOO interventions, supports a beneficial role for olive phenolics in cognitive health but does not yet allow attribution to individual biophenols. Future research should prioritise bioavailability-enhancing formulations, standardised compound characterisation, physiologically relevant dosing, and well-designed clinical trials targeting early-stage disease or preventive contexts.

Oleuropein and its aglycone, hydroxytyrosol, and oleocanthal demonstrate consistent preclinical efficacy across models of Alzheimer’s and Parkinson’s diseases through antioxidant and anti-inflammatory signalling, mitochondrial protection, autophagy enhancement, and modulation of amyloid and tau aggregation. Despite these multimodal actions, translation to human application is constrained by poor bioavailability, extensive metabolism, limited blood–brain barrier penetration, and methodological heterogeneity. Future directions focused on improved formulations, standardised compound characterisation, physiologically relevant dosing, and well-designed early stage clinical trials to bridge the gap between preclinical promise and clinical relevance.

Addressing these challenges will be essential to unlock the full therapeutic potential of olive biophenols in neurodegenerative diseases.

## Figures and Tables

**Table 1:** Characteristics of the included studies.

Study	Study Design	Component	Model	Disease Context	Risk of Bias
Abdallah et al., 2023 [29]	In vivo (mouse)	OC	OC vs. OC-low EVOO	AD	Moderate
Abuznait et al., 2013 [30]	In vitro + in vivo	OC	BBB & mouse models	AD	Moderate
Achour et al., 2016 [31]	In vitro (cellular)	OLE	Dopaminergic neuronal cells	PD	Moderate
Basellini et al., 2025 [32]	In vitro & in vivo	Oleuropein aglycone	α-synuclein PD models	PD	Moderate
Batarseh et al., 2017 [33]	In vitro (cellular)	OC	Astrocytes & neurons	AD	Moderate
Beauchamp et al., 2005 [34]	In vitro	OC	Enzymatic assays	Inflammation-related diseases; implications discussed for cardiovascular disease, cancer, platelet aggregation, and AD	Moderate
Brunetti et al., 2020 [28]	In vivo (*C. elegans*)	Oleuropein aglycone	*C. elegans*	Parkinson’s-like phenotypes	Low–Moderate
Brunetti et al., 2020 [28]	In vivo (*C. elegans*)	HT	*C. elegans*	Neurodegeneration & ageing	Low–Moderate
Funakohi-Tago et al., 2018 [35]	In vitro (cellular)	HT-butyrate	SH-SY5Y + 6-OHDA	PD	Moderate
Grossi et al., 2013 [36]	In vivo (transgenic mouse)	Oleuropein aglycone	TgCRND8 mice	AD (Aβ pathology)	Moderate
Li et al., 2009 [37]	In vitro (biochemical)	OC	Tau fibrillisation assays	AD	Moderate
Luccarini et al., 2015 [38]	In vivo & biochemical	Oleuropein aglycone	pE3-Aβ toxicity models	AD	Moderate
Monti et al., 2012 [39]	In vitro (biochemical)	OC	Tau aggregation assays	AD	Moderate
Nardiello et al., 2018 [40]	In vivo (mouse)	HT	Aβ deposition model	AD	Moderate
Pantano et al., 2017 [41]	In vivo (mouse)	Oleuropein aglycone	Cognitive & neuropathology models	AD	Moderate
Peng et al., 2016 [42]	In vivo (transgenic mouse)	HT	APP/PS1 mice	AD	Moderate
Qin et al., 2021 [43]	In vivo (transgenic mouse)	HT-acetate	APP/PS1 mice	AD	Moderate
Qosa et al., 2015 [44]	In vivo (transgenic mouse)	OC	TgSwDI mice	AD	Moderate
Singh et al., 2023 [45]	In vivo (toxin-induced)	OLE	Rotenone-induced rat model	PD	Low–Moderate
Sirangelo et al., 2020 [46]	In vitro (biochemical)	HT	Protein aggregation assays	Amyloid-related pathology	Moderate
Tajmim et al., 2021 [47]	In vivo (transgenic mouse)	OC	5xFAD mice	AD	Moderate
Visioli et al., 2022 [48]	In vitro (cellular)	HT	Neuronal AD cellular model	AD	Moderate
Yang et al., 2023 [49]	In vivo (mouse)	OC	Metabolic–behavioural AD model	AD	Moderate
Zhao et al., 2021 [50]	In vivo (mouse)	HT	Chronic stress model	Depression/neuroinflammation	Moderate
Zheng et al., 2015 [51]	In vivo (mouse)	HT	db/db mice	Metabolic cognitive impairment	Moderate

Note: AD—Alzheimer’s disease; PD—Parkinson’s disease; BBB—blood–brain barrier; EVOO—extra virgin olive oil; HT—hydroxytyrosol; OC—oleocanthal; OLE—oleuropein; α-syn—alpha-synuclein; Aβ—amyloid beta; APP/PS1—amyloid precursor protein presenilin 1 transgenic model; TgCRND8—transgenic mouse model overexpressing mutant amyloid precursor protein; TgSwDI—Swedish Dutch Iowa mutant amyloid precursor protein mouse model; 5xFAD—five familial Alzheimer’s disease mutation mouse model; 6-OHDA—6 hydroxydopamine; *C. elegans*—*Caenorhabditis elegans*.

**Table 2:** Mechanistic characteristics of included studies.

Study	Component	Main Mechanisms Investigated	Dose Range (Reported)	Route of Administration	Species/Model	Duration	Primary Outcome Measures
Abdallah et al., 2023 [29]	OC	Anti-amyloid; EVOO comparison	~10 mg/kg/day	Oral	AD mouse model	4 weeks	Aβ load, inflammation
Abuznait et al., 2013 [30]	OC	Aβ clearance; BBB transport	~10 mg/kg/day	Oral	AD mouse & BBB models	2 weeks	Aβ clearance
Achour et al., 2016 [31]	OLE	Mitochondrial protection; autophagy modulation; oxidative stress reduction	1–50 µM	Cell treatment	Dopaminergic cell line (PD model)	24–72 h	Cell viability, ROS, autophagy markers
Basellini et al., 2025 [32]	Oleuropein aglycone	Inhibition of α-synuclein aggregation; neuroprotection	1–20 µM; dietary equivalent in vivo	Cell treatment; oral	PD cellular & animal models	Acute–chronic	α-Syn aggregation, neuronal survival
Batarseh et al., 2017 [33]	OC	Protection against Aβ oligomer toxicity	1–10 µM	Cell treatment	Astrocytes & neurons	24–72 h	Cell viability
Beauchamp et al., 2005 [34]	OC	COX-1/2 inhibition (ibuprofen-like)	Dietary levels	Oral (EVOO)	Human sensory/biochemical	Acute	COX inhibition
Brunetti et al., 2020 [28]	Oleuropein aglycone	Stress resistance; proteostasis; longevity pathways	~50 µM	Feeding	*C. elegans* PD-like model	Lifespan	Motor function, lifespan, aggregation
Brunetti et al., 2020 [28]	HT	Proteostasis; longevity; stress resistance	~50 µM	Feeding	*C. elegans*	Lifespan	Motor activity, survival
Fuccelli et al., 2018 [52]	HT	Anti-inflammatory; antioxidant	10–50 mg/kg/day	Oral	Mouse systemic inflammation model	7 days	Cytokines, oxidative stress
Funakohi-Tago et al., 2018 [35]	HT butyrate	Nrf2/HO-1 activation; anti-apoptotic	1–20 µM	Cell treatment	SH-SY5Y (6-OHDA PD model)	24 h	Apoptosis, antioxidant enzymes
Grossi et al., 2013 [36]	Oleuropein aglycone	Anti-amyloidogenic; reduction in Aβ aggregation and plaque burden; autophagy induction	~50 mg/kg/day	Diet supplementation	TgCRND8 mice (AD)	8 weeks	Aβ plaque load, synaptic integrity, cognition
Li et al., 2009 [37]	OC	Tau fibrillisation inhibition	1–10 µM	Biochemical assay	Tau aggregation system	Acute	Tau fibril formation
Luccarini et al., 2015 [38]	Oleuropein aglycone	Protection against pE3-Aβ toxicity; epigenetic modulation; synaptic preservation	10–50 µM (in vitro); dietary equivalent in vivo	Cell treatment; diet	Neuronal cultures; mouse models	Acute–chronic	Neuronal viability, epigenetic markers, synaptic function
Monti et al., 2012 [39]	OC	Tau fibrillisation modulation	1–20 µM	Biochemical assay	Tau protein system	Acute	Tau aggregation kinetics
Nardiello et al., 2018 [40]	HT	Anti-amyloid; synaptic restoration	~50 mg/kg/day	Oral	Aβ-depositing mouse model	8 weeks	Aβ load, cognition
Pantano et al., 2017 [41]	Oleuropein aglycone	Anti-amyloid; antioxidant; cognitive rescue	~5–50 mg/kg/day (polyphenol-rich extract)	Oral (diet)	AD-like mouse model	8 weeks	Cognitive performance, Aβ pathology
Peng et al., 2016 [42]	HT	Cognitive improvement independent of APP processing	5–50 mg/kg/day	Oral	APP/PS1 mice	6 months	Cognitive tests, synaptic markers
Qin et al., 2021 [43]	HT-acetate	ERβ-dependent neuroprotection; synaptic plasticity	~30 mg/kg/day	Oral	APP/PS1 mice	12 weeks	Learning, memory, ERβ signalling
Qosa et al., 2015 [44]	OC	Enhanced Aβ brain clearance	~10 mg/kg/day	Oral	TgSwDI mice	4 weeks	Brain Aβ levels
Singh et al., 2023 [45]	OLE	Antioxidant; BDNF/CREB/Akt signalling; mitochondrial protection	25–100 mg/kg/day	Oral gavage	Rotenone-induced PD rat model	28 days	Motor behaviour, dopaminergic neuron survival
Sirangelo et al., 2020 [46]	HT	Inhibition of protein oligomerisation	10–100 µM	Biochemical assay	Human insulin aggregation model	Acute	Amyloid fibril formation
Tajmim et al., 2021 [47]	OC	Anti-amyloid; oral bioavailability	5–20 mg/kg/day	Oral formulation	5xFAD mice	3 months	Aβ pathology, cognition
Visioli et al., 2022 [48]	HT	Mitochondrial energetics enhancement	1–10 µM	Cell treatment	AD-related neuronal cells	24–48 h	ATP production, respiration
Yang et al., 2023 [49]	OC	Metabolic modulation; behavioural improvement	10 mg/kg/day	Oral	AD mouse model	8 weeks	Behaviour, metabolic markers
Zhao et al., 2021 [50]	HT	Anti-inflammatory; neurotrophic signalling	20–80 mg/kg/day	Oral	Stress-induced mouse model	4 weeks	Behaviour, cytokines, BDNF
Zheng et al., 2015 [51]	HT	AMPK activation; mitochondrial bioenergetics; antioxidant	~10 mg/kg/day	Oral	db/db mice	8 weeks	Mitochondrial function, oxidative stress

Notes: AD—Alzheimer’s disease; PD—Parkinson’s disease; Aβ—amyloid beta; α-syn—alpha-synuclein; BBB—blood–brain barrier; HT—hydroxytyrosol; OC—oleocanthal; EVOO—extra virgin olive oil; ROS—reactive oxygen species; ATP—adenosine triphosphate; COX—cyclooxygenase; Nrf2—nuclear factor erythroid 2-related factor 2; HO-1—heme oxygenase 1; ERβ—estrogen receptor beta; BDNF—brain-derived neurotrophic factor; OLE—oleuropein; CREB—cAMP response element binding protein; Akt—protein kinase B; AMPK—adenosine monophosphate activated protein kinase; pE3-Aβ—pyroglutamylated amyloid beta; *C. elegans*—*Caenorhabditis elegans*.

**Table 3:** Key findings, strength of evidence, and translational barriers of included studies.

Study	Component	Key Findings	Strength & Consistency of Evidence	Key Translational Barriers
Abdallah et al., 2023 [29]	OC	Superior efficacy of purified oleocanthal vs. EVOO	Moderate	Standardisation and dose scalability
Abuznait et al., 2013 [30]	OC	Enhanced Aβ clearance across BBB and in vivo	Moderate–strong (combined in vitro/in vivo)	Long-term safety and dosing in humans are unknown
Achour et al., 2016 [31]	OLE	Reduced mitochondrial ROS and modulation of autophagy in dopaminergic cells	Limited–moderate (cellular model only)	Lack of in vivo confirmation and pharmacokinetics
Basellini et al., 2025 [32]	Oleuropein aglycone	Inhibited α-synuclein aggregation and protected neurons across PD models	Moderate (multi-model consistency)	The human relevance of aggregation inhibition remains uncertain
Batarseh et al., 2017 [33]	OC	Protection against Aβ oligomer toxicity in neural cells	Limited–moderate	Lack of in vivo behavioural data
Beauchamp et al., 2005 [34]	OC	COX inhibition comparable to ibuprofen	Strong (mechanistic)	Not disease-specific; indirect neuroprotection
Brunetti et al., 2020 [28]	Oleuropein aglycone	Improved proteostasis, motor function, and lifespan in *C. elegans*	Limited–moderate (ageing model relevance)	Evolutionary distance; dose translation unclear
Brunetti et al., 2020 [28]	HT	Enhanced stress resistance and lifespan in *C. elegans*	Limited–moderate	Model simplicity and translational uncertainty
Fuccelli et al., 2018 [52]	HT	Reduced systemic inflammation and oxidative stress	Limited–moderate (indirect neuroprotection)	Not disease-specific to neurodegeneration
Funakohi-Tago et al., 2018 [35]	HT-butyrate	Protection against 6-OHDA-induced apoptosis via Nrf2/HO-1	Limited–moderate (pathway specificity)	In vitro PD model limits translational inference
Grossi et al., 2013 [36]	Oleuropein aglycone	Reduced Aβ plaque burden, improved synaptic integrity and cognition in TgCRND8 mice	Moderate–strong (robust in vivo AD model, consistent outcomes)	Limited pharmacokinetic data; dietary dose equivalence to humans unclear
Li et al., 2009 [37]	OC	Direct inhibition of tau fibrillisation	Moderate (clear molecular target)	Requires validation in living systems
Luccarini et al., 2015 [38]	Oleuropein aglycone	Protection against pE3-Aβ toxicity; epigenetic modulation and synaptic preservation	Moderate (mechanistic depth, mixed in vitro/in vivo)	Translational relevance of epigenetic effects not fully established
Monti et al., 2012 [39]	OC	Modulation of tau fibrillisation kinetics	Limited	Biochemical model only
Nardiello et al., 2018 [40]	HT	Reduced Aβ deposition and restored cognitive function	Moderate (well-characterised AD mouse model)	Brain bioavailability is not directly measured
Pantano et al., 2017 [41]	Oleuropein aglycone	Improved cognition and reduced neuropathology following polyphenol supplementation	Moderate (in vivo efficacy, extract complexity)	Contribution of oleuropein aglycone vs. other polyphenols not isolated
Peng et al., 2016 [42]	HT	Mild cognitive improvement independent of APP processing	Moderate (long-term in vivo exposure)	Limited mechanistic linkage to amyloid pathology
Qin et al., 2021 [43]	HT-acetate	Cognitive improvement mediated by ERβ signalling	Moderate (clear receptor-dependent mechanism)	Ester derivative relevance to dietary HT uncertain
Qosa et al., 2015 [44]	OC	Increased brain Aβ clearance in TgSwDI mice	Moderate–strong (consistent with prior work)	Translational relevance of clearance magnitude
Singh et al., 2023 [45]	OLE	Neuroprotection in PD model via BDNF/CREB/Akt signalling	Moderate (clear pathway activation, toxin-induced model)	The acute toxin model may not reflect progressive PD pathology
Sirangelo et al., 2020 [46]	HT	Inhibited amyloid oligomerisation in biochemical assays	Limited (biochemical evidence only)	Absence of cellular or in vivo confirmation
Tajmim et al., 2021 [47]	OC	Reduced amyloid pathology and improved cognition with oral formulations	Moderate–strong (formulation + in vivo efficacy)	Human pharmacokinetics are still unresolved
Visioli et al., 2022 [48]	HT	Enhanced mitochondrial energetics in AD-related cells	Limited–moderate (cellular mechanistic evidence)	Lack of behavioural or in vivo validation
Yang et al., 2023 [49]	OC	Improved metabolic and behavioural phenotypes in AD mice	Moderate	Indirect mechanism; metabolic confounding
Zhao et al., 2021 [50]	HT	Reduced neuroinflammation and improved stress-related behaviours	Moderate (in vivo behavioural relevance)	Applicability to neurodegenerative disease unclear
Zheng et al., 2015 [51]	HT	Improved brain mitochondrial function via AMPK activation	Moderate (in vivo metabolic–neural link)	Disease specificity to AD/PD indirect

Notes: AD—Alzheimer’s disease; PD—Parkinson’s disease; Aβ—amyloid beta; BBB—blood–brain barrier; EVOO—extra virgin olive oil; HT—hydroxytyrosol; OC—oleocanthal; ROS—reactive oxygen species; ERβ—estrogen receptor beta; BDNF—brain-derived neurotrophic factor; CREB—cAMP response element binding protein; Akt—protein kinase B; AMPK—adenosine monophosphate activated protein kinase; Nrf2—nuclear factor erythroid 2-related factor 2; HO-1—heme oxygenase 1.

## Data Availability

No new data were created or analysed in this study.

## Abbreviations

The following abbreviations are used in this manuscript:

5xFAD	Five familial Alzheimer’s disease mutation mouse model
6-OHDA	6-hydroxydopamine
α-syn or αSN	Alpha-synuclein
AD	Alzheimer’s disease
Akt	Protein kinase B
AMPK	Adenosine monophosphate activated protein kinase
APP/PS1	Amyloid precursor protein and presenilin 1 transgenic mouse model
APP	Amyloid precursor protein
ATP	Adenosine triphosphate
Aβ	Amyloid beta
BBB	Blood–brain barrier
BDNF	Brain-derived neurotrophic factor
C. elegans	Caenorhabditis elegans
COX	Cyclooxygenase
CREB	cAMP response element binding protein
ERβ	Estrogen receptor beta
EVOO	Extra virgin olive oil
HO-1	Heme oxygenase 1
HT	Hydroxytyrosol
M-OHAT	Modified Office for Health Assessment and Translation
Nrf2	Nuclear factor erythroid 2-related factor 2
OC	Oleocanthal
OLE	Oleuropein
PD	Parkinson’s disease
pE3-Aβ	Pyroglutamylated amyloid beta
PRISMA	Preferred Reporting Items for Systematic Reviews and Meta-Analyses
PROSPERO	Prospective Register of Systematic Reviews
RoB	Risk of bias
ROS	Reactive oxygen species
SYRCLE	Systematic Review Centre for Laboratory Animal Experimentation
TgCRND8	Transgenic mouse model overexpressing mutant amyloid precursor protein
TgSwDI	Swedish Dutch Iowa mutant amyloid precursor protein mouse model
ToxR	Toxicological data reliability
